# Decentralized control scheme for myriapod robot inspired by adaptive and resilient centipede locomotion

**DOI:** 10.1371/journal.pone.0171421

**Published:** 2017-02-02

**Authors:** Kotaro Yasui, Kazuhiko Sakai, Takeshi Kano, Dai Owaki, Akio Ishiguro

**Affiliations:** 1 Research Institute of Electrical Communication, Tohoku University, Sendai, Miyagi, Japan; 2 Japan Science and Technology Agency, CREST, Kawaguchi, Saitama, Japan; Nanjing University, CHINA

## Abstract

Recently, myriapods have attracted the attention of engineers because mobile robots that mimic them potentially have the capability of producing highly stable, adaptive, and resilient behaviors. The major challenge here is to develop a control scheme that can coordinate their numerous legs in real time, and an autonomous decentralized control could be the key to solve this problem. Therefore, we focus on real centipedes and aim to design a decentralized control scheme for myriapod robots by drawing inspiration from behavioral experiments on centipede locomotion under unusual conditions. In the behavioral experiments, we observed the response to the removal of a part of the terrain and to amputation of several legs. Further, we determined that the ground reaction force is significant for generating rhythmic leg movements; the motion of each leg is likely affected by a sensory input from its neighboring legs. Thus, we constructed a two-dimensional model wherein a simple local reflexive mechanism was implemented in each leg. We performed simulations by using this model and demonstrated that the myriapod robot could move adaptively to changes in the environment and body properties. Our findings will shed new light on designing adaptive and resilient myriapod robots that can function under various circumstances.

## Introduction

Robots are now required to perform adequately under unpredictable and unstructured environments such as those in disaster areas. Therefore, researchers have drawn inspiration from legged animals that can move adaptively and resiliently under various circumstances, and many legged robots have been developed thus far. Although most of these studies have focused on animals with a few legs; for example, bipeds, quadrupeds, and hexapods [1, 2]; myriapods have recently attracted attention [3–8] because myriapod robots are expected to have higher stability and fault tolerance than robots with a few legs.

However, the development of myriapod robots is not easy because of the following reasons. Myriapod robots have numerous legs that are difficult to coordinate in real time. Several researchers used control schemes in which the phase relationship among the legs is fixed [3–5], yet this approach has a limitation: adaptability. In fact, it is completely different from that of real myriapods that exhibit adaptive locomotion by changing the phase relationship among the legs in response to the circumstances encountered in real time [9].

The key to solve this problem is autonomous decentralized control in which the nontrivial macroscopic behavior of an entire system emerges through the coordination of simple individual components. Accordingly, several decentralized control schemes for myriapod robots have been proposed. For example, Inagaki et al. [6] proposed a decentralized and event-driven control scheme for centipede-like multilegged robots to walk on uneven terrain. Matthey et al. [7] designed a decentralized control mechanism based on central pattern generators (CPGs), which are neural networks capable of producing coordinated patterns of rhythmic activity [10]. They then investigated its validity via simulations. Further, Onat et al. [8] demonstrated a multilegged robot for which a stable gait pattern was developed using a decentralized control mechanism in which each leg’s ground contact signals are exploited. Although the control schemes proposed in these studies contributed to the adaptability enhancement of myriapod robots, their behavior is still much less adaptive than that of real myriapods.

Therefore, we considered the behavior of centipedes in this study. Centipedes can walk under unstructured environments adaptively via coordination of their numerous legs. They achieve this locomotion by reasonably propagating leg-density waves along the body axis [11]. It is known that this form of leg-density waves adaptively vary according to circumstances such as change in walking speed [9]. Hence, there likely exists an ingenious decentralized control mechanism that could be used for designing highly adaptive myriapod robots.

In this study, we aim to design a decentralized control scheme for myriapod robots by drawing inspiration from centipede locomotion. For this purpose, we adopted a minimalist approach. Specifically, we propose a simple phenomenological model on the basis of behavioral experiments to effectively extract the essence of the control mechanism. We performed two experiments using centipedes. First, we observed the response when part of the terrain was removed during locomotion. Second, we observed the locomotion of leg-amputated centipedes. These experiments showed that periodic leg movement is not generated without ground contact, and motion of a leg is likely affected by sensory input from itself and its neighboring legs. Accordingly, we constructed a mathematical model that implements a simple decentralized control scheme on the basis of these findings. We performed simulations by using the proposed control scheme and succeeded in reproducing some qualitative characteristics of centipede locomotion.

## Behavioral experiments

**Fig 1:**
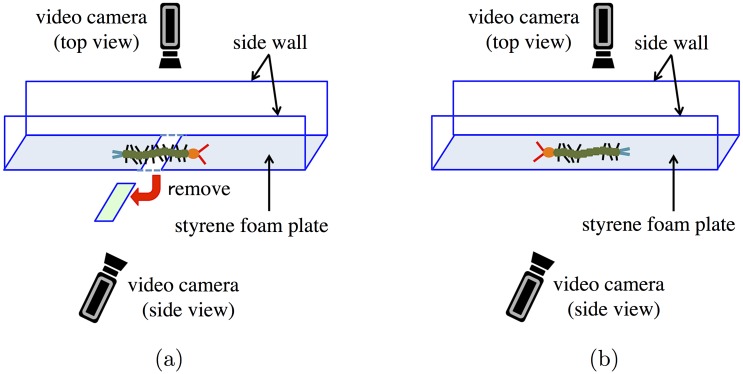
Schematic of the experimental set up. (a) Experiment in which part of the terrain was removed during locomotion. (b) Experiment in which a few legs of centipedes were amputated.

The adaptive and resilient locomotion in animals is said to be generated through close interaction between the nervous system, body, and environment [12]; centipede locomotion is likely no exception. Observations of changes in qualitative behaviors according to changes in the environment or body properties enable us to extract the essence of the underlying control mechanism. Hence, we performed two behavioral experiments. First, we observed the responses of centipedes when part of the terrain was removed during their locomotion (Fig 1(a)). Second, we observed the locomotion of leg-amputated centipedes to investigate the effects of changes in body properties (Fig 1(b)).

We used three adult centipedes (*Scolopendra subspinipes*) whose body lengths along the body axis range from 14 to 16 cm and recorded five trials for each experiment. In both experiments, we observed forward locomotion on a flat styrene foam plate and recorded the top and side views by using high-speed video cameras (DITECT, type HAS-L1) at a resolution of 640×480 pixels and 300 fps (Fig 1). To analyze the observed behavior, we visualized leg-tip positions with spatiotemporal plots using the image processing package ImageJ [13]. More specifically, we obtained spatiotemporal plots as follows: we first drew suitable lines on the top-view images and then sequentially arranged the images cropped along the line.

**Fig 2:**
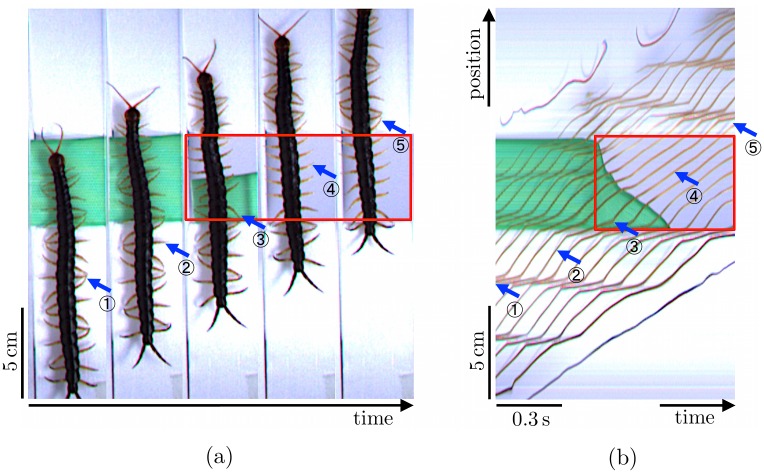
Results for the experiment in which part of the terrain was removed during locomotion. (a) Photographs taken approximately every 0.3 s. (b) Spatiotemporal plot of leg-tip positions. One of the legs on the right-hand side is denoted by blue arrows; the same numbers indicate the leg-tip position of a certain leg at the same time. Red squares denote areas wherein legs do not contact the ground. Legs stop periodic movement in these areas.

In the first experiment, we removed a part of the terrain, with a length 5 cm, during locomotion (Fig 1(a) and S1 Movie). Fig 2(a) and 2(b) show photographs of one of the centipedes during its locomotion and the spatiotemporal plot of its leg-tip positions, respectively. We determined that the leg tips form density waves that propagate backward at the commencement of the experiment, as reported in previous studies [9]. However, the behavioral changes immediately after a part of the terrain is removed show that legs over the gap stop periodical movement and remain at certain positions while the other legs continue to move periodically. The resting legs start to move again when their nearest anterior legs touch the ground. All the centipedes exhibit similar behaviors in all trials.

**Fig 3:**
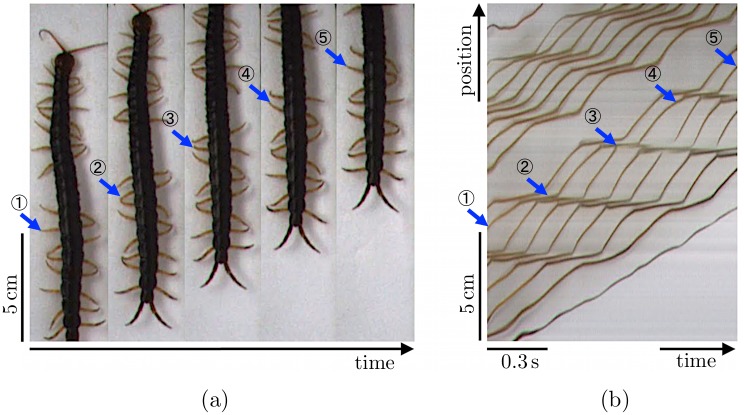
Results for the experiment in which centipedes’ legs were amputated. (a) Photographs taken approximately every 0.3 s. (b) Spatiotemporal plot of leg-tip positions. One of the legs on the left-hand side is denoted by blue arrows; the same numbers indicate the leg-tip position of a certain leg at the same time. The leg-amputated centipede walks by propagating leg density waves backward at both anterior and posterior sides of the leg-amputated region.

In the second experiment, we observed the centipede locomotion, in which three successive pairs of legs at the middle part of the body were amputated (Fig 1(b) and S2 Movie). Fig 3(a) and 3(b) show photographs of one of the centipedes during its locomotion and the spatiotemporal plot of its leg-tip positions, respectively. The leg-amputated centipede can walk by propagating leg density waves backward at both anterior and posterior sides of the leg-amputated region. All the centipedes exhibit similar behaviors for all trials.

## Mathematical model

**Fig 4:**
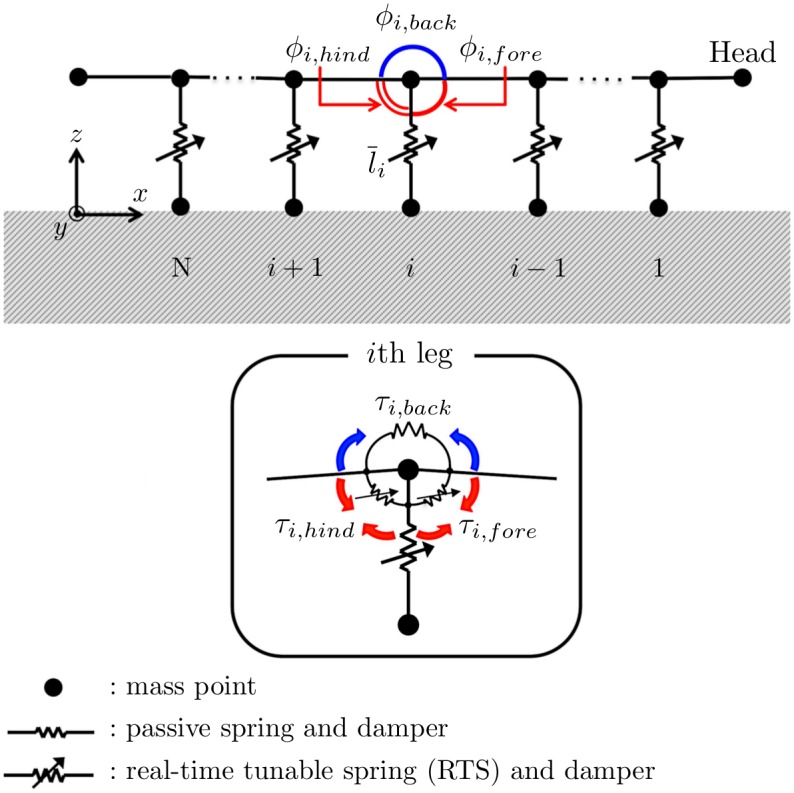
Schematic of the physical model.

We propose a mathematical model based on the aforementioned behavioral experiments. As we mainly focus on the coordination mechanism between legs along the body axis, we simply modeled the body two-dimensionally, that is, each segment has only one leg (Fig 4). The body trunk consists of mass points connected one-dimensionally through rigid links. Each leg has a mass at its tip and is connected to the body trunk using a parallel combination of a real-time tunable spring (RTS) and a damper. The RTS is a spring whose natural length can be actively changed [14]. The detailed model for interaction between the leg tips and ground is described in the supporting information (see S1 Appendix).

Three pairs of torsional springs and dampers are embedded at the proximal end of each leg. Two of these springs connected to the leg are torsional springs with natural angles that can be actively changed; thus, they can be used as actuators. The other torsional spring, which is implemented on the body trunk, is passive with a natural angle of *π*; this prevents the body trunk from bending excessively. Thus, the torques *τ*_*i*,*fore*_, *τ*_*i*,*hind*_, and *τ*_*i*,*back*_ acting on the *i*th proximal joint (as defined in Fig 4) are described as

$$\tau_{i,back}=k_{1}\left(\phi_{i,back}-\pi \right)+d_{1}{\dot{\phi }}_{i,back}$$ (1)

$$\tau_{i,fore}=k_{2}\left(\phi_{i,fore}-{\bar{\phi }}_{i,fore}\right)+d_{2}{\dot{\phi }}_{i,fore}$$ (2)

$$\tau_{i,hind}=k_{2}\left(\phi_{i,hind}-{\bar{\phi }}_{i,hind}\right)+d_{2}{\dot{\phi }}_{i,hind},$$ (3)

where *k*_1_ and *k*_2_ are the spring constants of the torsional springs; *d*_1_ and *d*_2_ are the damping coefficients; *ϕ*_*i*,*back*_, *ϕ*_*i*,*fore*_, and *ϕ*_*i*,*hind*_ (as defined in Fig 4) are the actual angles of the *i*th joint; and ${\bar{\phi }}_{i,fore}$ and ${\bar{\phi }}_{i,hind}$ are the target angles of the *i*th joint.

The behavioral experiments showed that the ground reaction force produced by the interaction between the legs and environment plays a significant role in generating rhythmic leg movement. In addition, the motion of a leg is likely affected by sensory input from its neighboring legs. Further, we considered that centipedes exploit horizontal rather than vertical components of the ground reaction force because the effect of propulsion is likely more important than that of body support owing to the large number of legs. Thus, we hypothesized that each leg is driven by a local reflexive mechanism in which the horizontal components of ground reaction forces acting on the corresponding leg and its adjacent legs are exploited. Accordingly, we modeled the target angles of the *i*th joint (${\bar{\phi }}_{i,fore}$ and ${\bar{\phi }}_{i,hind}$) and the natural length of the *i*th RTS $\bar{l_{i}}$ as

$$\lambda {\dot{\bar{\phi }}}_{i,fore}=\frac{\pi }{2}+\alpha_{1}S_{i}-\alpha_{2}\sum_{j=i\pm 1}S_{j}-{\bar{\phi }}_{i,fore},$$ (4)

$$\lambda {\dot{\bar{\phi }}}_{i,hind}=\frac{\pi }{2}-\alpha_{1}S_{i}+\alpha_{2}\sum_{j=i\pm 1}S_{j}-{\bar{\phi }}_{i,hind},$$ (5)

$$\lambda {\dot{\bar{l}}}_{i}=l_{0}+\beta_{1}S_{i}-\beta_{2}\sum_{j=i\pm 1}S_{j}-{\bar{l}}_{i},$$ (6)

where

$$S_{i}=\tanh\left(aF_{i}\right),$$ (7)

with *α*_1_, *α*_2_, *β*_1_, *β*_2_, *λ*, *l*_0_, and *a* being positive constants. Here, *F*_*i*_ is the horizontal component of the ground reaction force acting on the *i*th leg, and the direction of movement is taken as positive. The mathematical formula of *F*_*i*_ is described in the supporting information (see S1 Appendix). The nondimensional quantity *S*_*i*_ characterizes the force sensation detected at the *i*th leg, where the hyperbolic tangent function is used because sensory information likely saturates with the increase in the ground reaction force.

**Fig 5:**
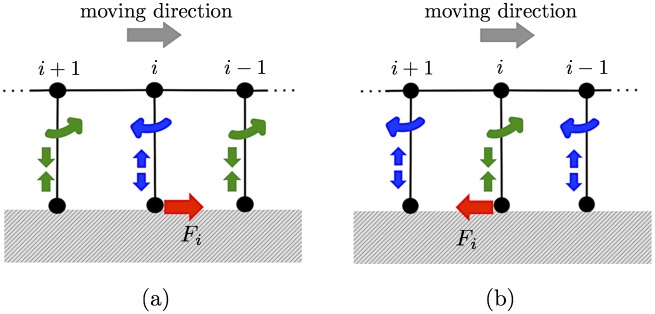
Effect of the local reflexive mechanism. (a) Response when the *i*th leg detects a ground reaction force beneficial for propulsion. (b) Response when the *i*th leg detects a ground reaction force that impedes propulsion.

The physical meaning of Eqs (4)–(7) is explained as follows. When the legs do not contact the ground, that is, *S*_*i*_ = 0 for all *i*, then ${\bar{\phi }}_{i,fore}={\bar{\phi }}_{i,hind}=\pi /2$ and ${\bar{l}}_{i}=l_{0}$; thus, legs are aligned perpendicular to the body trunk and do not move. When legs contact the ground, they begin to move because of the local reflexive mechanism described in the second and third terms on the right-hand side of Eqs (4)–(6). The second term denotes a reflex that acts on a leg detecting ground reaction force. In particular, when a leg detects a ground reaction force beneficial for propulsion, it reflexes by kicking the ground backward to further attain the beneficial ground reaction force (Fig 5(a)). In contrast, when a leg detects a ground reaction force that impedes propulsion, it reflexes by throwing itself forward by lifting its leg tip off the ground to reduce the undesirable ground reaction force (Fig 5(b)). The third term denotes a reflex to a force detected at adjacent legs. In other words, when a leg detects a ground reaction force, the reflex is on its adjacent legs. More specifically, when a leg detects a ground reaction force beneficial for propulsion, the adjacent legs throw themselves forward by lifting their tips off the ground (Fig 5(a)). Moreover, when a leg detects a ground reaction force that impedes propulsion, the adjacent legs kick the ground backward (Fig 5(b)). Thus, it is expected that neighboring legs do not interfere with each other for the body to propel itself effectively.

## Simulation results

We conducted simulations to verify the validity of the proposed model; the program was written in C++ and simulation results were visualized using OpenGL. The differential equations were solved using the fourth-order Runge–Kutta method with a time step of 1.234×10^−5^ s. Table 1 lists the parameter values employed in the simulations, chosen by trial and error, and Table 2 shows the initial conditions.

**Table 1:** Parameter values employed in the simulations.

Parameter	Value	Dimension
Number of legs	20	
Weight of each body trunk mass	3.08×10^−4^	[kg]
Weight of head mass	3.08×10^−4^	[kg]
Weight of tail mass	3.08×10^−4^	[kg]
Weight of each leg tip mass	6.15×10^−5^	[kg]
Spring constant of rigid springs	4.04×10^2^	[kgs^−2^]
Natural length of rigid springs	0.76×10^−2^	[m]
Damping coefficient of rigid links	4.99	[kgs^−1^]
Spring constant of the RTS	4.04	[kgs^−2^]
*l*_0_	0.76×10^−2^	[m]
Damping coefficient of leg links	4.99×10^−2^	[kgs^−1^]
*k*_1_	7.50×10^−2^	[m^2^s^−2^kg]
*k*_2_	3.75×10^−4^	[m^2^s^−2^kg]
*d*_1_	3.47×10^−5^	[m^2^s^−1^kg]
*d*_2_	1.16×10^−6^	[m^2^s^−1^kg]
*α*_1_	0.32	
*α*_2_	0.16	
*β*_1_	1.83×10^−3^	[m]
*β*_2_	0.91×10^−3^	[m]
*λ*	2.47×10^−3^	[s]
*a*	4.88×10^2^	[m^−1^kg^−1^s^2^]
*k*_*g*_	3.23×10^1^	[kgs^−2^]
*c*_*g*_	4.99×10^−3^	[kgs^−1^]
*c*	0.89×10^2^	[m^−1^s]
*μ*	2	

The definitions of *k*_*g*_, *c*_*g*_, *c*, and *μ* are provided in the supporting information (S1 Appendix).

**Table 2:** Initial conditions for the simulations.

	Value	Dimension
*x* coordinate of head mass	0.16	[m]
*x* coordinate of tail mass	0	[m]
*x* coordinate of *i*th leg tip mass	0.16 − 0.0076*i*	[m]
*z* coordinate of *i*th leg tip mass	0	[m]
*ϕ*_*i*,*fore*_, *ϕ*_*i*,*hind*_, ${\bar{\phi }}_{i,fore}$, ${\bar{\phi }}_{i,hind}$	*π*/2	
*ϕ*_*i*,*back*_	*π*	

**Fig 6:**
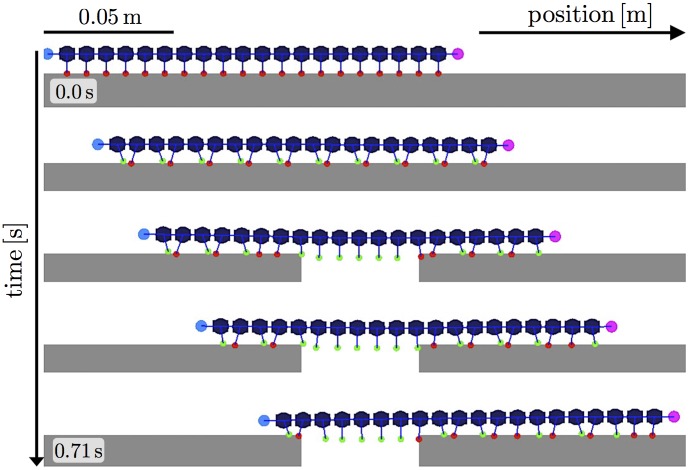
Snapshots of the simulated myriapod robot. Part of the terrain was removed during locomotion. A snapshot was taken approximately every 0.18 s. Legs with tips on and off the ground are colored in red and green, respectively.

**Fig 7:**
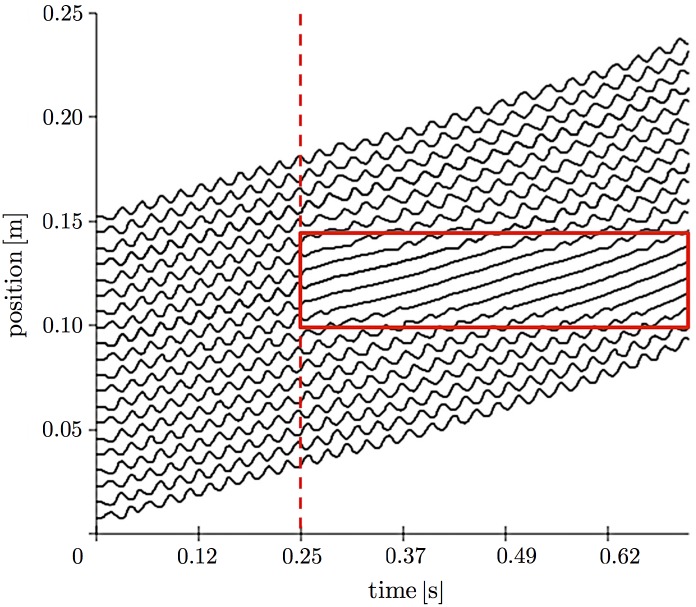
Spatiotemporal plots of leg positions in the simulation in which part of the terrain was removed. Red dashed line denotes the time when part of the terrain was removed. Red squares denote areas where the legs do not contact the ground.

First, we examined the response of locomotion to changes in the environment by setting the simulation condition to be equivalent to that in the first behavioral experiment (Fig 1(a)). More specifically, a part of the terrain was removed at 0.247 s, and a change in locomotion pattern was then observed. Figs 6 and 7 show the snapshots and a spatiotemporal plot of leg-tip positions along the horizontal axis, respectively. At the beginning of the experiment, the simulated myriapod robot propagated leg density waves backward, although the wave number was higher than that for real centipedes. After a part of the terrain was removed, the legs over the gap stopped periodical movement and remained at a neutral position, while the other legs continued to move periodically. This behavior is qualitatively similar to the locomotion of real centipedes observed in our behavioral experiment (Fig 2 and S1 Movie).

**Fig 8:**
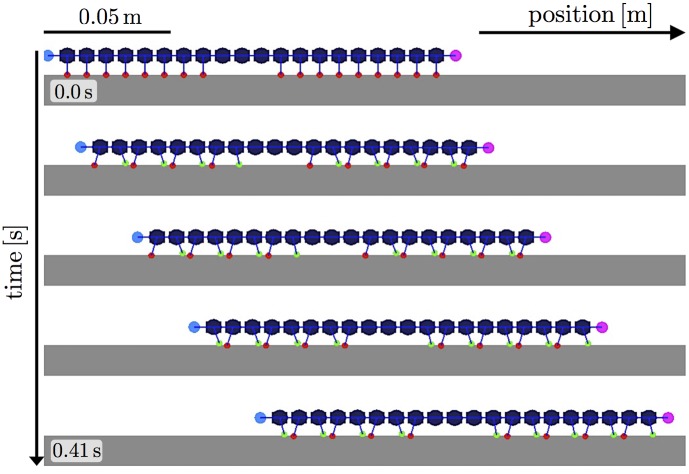
Snapshots of a simulated myriapod robot with leg amputations. Each snapshot was taken approximately every 0.10 s. Legs with tips on and off the ground are colored by red and green, respectively.

**Fig 9:**
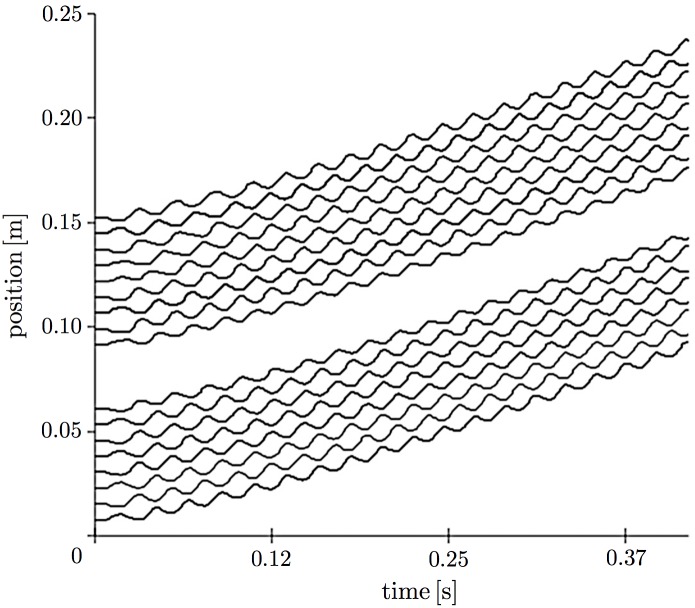
Spatiotemporal plot of leg positions of a simulated myriapod robot with leg amputations.

Next, we examined the locomotion of a leg-amputated centipede on a flat terrain. We removed three successive pairs of legs in the middle part of the body. Figs 8 and 9 show that the simulated myriapod robot could walk by propagating leg density waves backward at both anterior and posterior sides of the leg-amputated part similar to real centipedes (Fig 3 and S2 Movie). Thus, our proposed model reproduced the findings of the behavioral experiments fairly well.

## Discussion

With the help of behavioral experiments on centipede locomotion and mathematical modeling, we developed a decentralized control scheme for the interlimb coordination of myriapod robots. Our proposed control scheme is based on a local reflexive mechanism in which each leg exploits horizontal ground reaction forces acting on itself and its neighboring legs. Despite the simplicity of the control scheme, the simulated myriapod robot successfully reproduced several qualitative findings of behavioral experiments on centipede locomotion. First, they walk by propagating leg density waves. Second, their legs stop periodical movement when they do not detect ground reaction forces. Third, when some of the legs are amputated, the remaining legs form density waves to walk. In particular, the second and third findings have not been realized by any model proposed previously [6–8]. Therefore, we consider that our control scheme paves the way for developing highly adaptive, energy-efficient, and resilient myriapod robots. Indeed, the energy consumed by the robot could be greatly reduced by stopping the motion of legs that are not in contact with the ground.

We believe that the adaptability will be further improved by extending our 2D model to 3D. However, our model described herein is advantageous in that it captures the essence of myriapod locomotion owing to its simplicity. Indeed, simple models generally help in understanding the essence of complicated phenomena, and it is not an exception in the field of animal locomotion (*e.g.* the model proposed by Maus et al. [15]). Furthermore, our model is significant enough because it would be possible to develop a real robot on the basis of our 2D model by restricting yaw and roll motion, as in our previous study on bipedal locomotion [16]. Experimental verification of our model using a real robot remains as a future work.

Several differences still exist between the behavioral experiments and simulation. For example, the wave number of the leg density waves formed in the simulation was much larger than that formed in real centipedes (Figs 2(b), 3(b), 7 and 9). This is probably because the neural system in centipedes is much more complicated than that considered in our model; although we assumed that the ground reaction force on each leg affects only itself and its adjacent legs, the force could affect more distant legs in a complicated manner for real centipedes. In addition, the interlimb coordination might be affected by motor commands from the central nervous system, while the control scheme in our model is fully decentralized. To deal with such problems, reconsidering the model from biological perspectives might be important. In fact, some detailed studies conducted on the interlimb coordination mechanism in hexapods showed how sensory information of a leg affects the behavior of the other legs on the basis of neurophysiological experiments [17]. Hence, further investigations including neurophysiological studies are clearly needed to understand the control mechanism underlying centipede locomotion.

## Supporting information

S1 Appendix Model for Interaction between the Legs and Ground. (PDF)

S1 Movie Centipede locomotion on terrain with a gap. (MP4)

S2 Movie Leg-amputated centipede’s locomotion. (MP4)

S3 Movie Simulated myriapod robot locomotion on terrain with a gap. (MP4)

S4 Movie Leg-amputated myriapod robot locomotion in the simulation. (MP4)
