# Supplementary material for: Decentralized control scheme for myriapod robot inspired by adaptive and resilient centipede locomotion
Source: PLoS One. 2017 Feb 2;12(2):e0171421. doi: 10.1371/journal.pone.0171421 (PMC5289600; doi:10.1371/journal.pone.0171421)
Supplement: S1 Appendix — (PDF) [file pone.0171421.s003.pdf]

Model for Interaction Between the Legs and the Ground

Kotaro Yasui[1], Kazuhiko Sakai[1], Takeshi Kano[1], Dai Owaki[1], Akio Ishiguro[1,2]

1 Research Institute of Electrical Communication, Tohoku University, Sendai, Miyagi, Japan
2 Japan Science and Technology Agency, CREST, Kawaguchi, Saitama, Japan

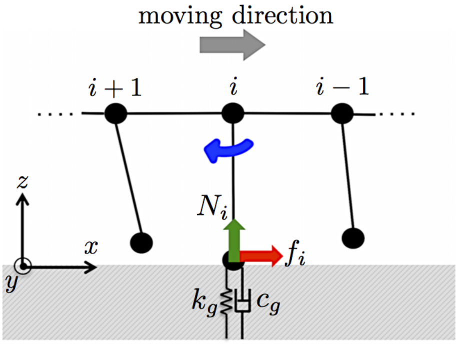

Figure 1. Schematic of the interaction between the legs and the ground.

As shown in Fig 1, we assumed that the ground has a viscoelastic property. The normal force acting on the ith leg tip N_i is described as

$$N_i = \max[-k_g z_i - c_g \dot{z}_i, 0]$$

where k_g is the spring constant and c_g is the damping coefficient.

On the other hand, we modeled friction forces using Coulomb friction. However, because it was difficult to treat static friction in simulations, we simply assumed that the frictional force, *i.e.*, the horizontal component of the ground reaction force, acting on the ith leg f_i is given as

$$f_i = -\mu N_i \tanh(c\dot{x}_i), \tag{1}$$

where μ is the friction coefficient and c is a positive constant. The hyperbolic tangent function is introduced to reproduce dynamic Coulomb friction. In fact, Eq. (1) accurately describes dynamic Coulomb friction when $|\dot{x}_i| \gg c^{-1}$. Thus, the force acting on the ith leg from the ground $\mathbf{F}_{\mathrm{reaction},i}$ is given by

$$\mathbf{F}_{\mathrm{reaction},i} = (f_i, N_i)^{\mathrm{T}}.$$

When part of the terrain was removed, the interaction between the ith leg tip and the side wall was modeled as follows (Fig 2). When $x_i < x_{\mathrm{gap,l}}$, $z_i < x_i - x_{\mathrm{gap,l}}$, and

$z_i < 0$ where $x_{\mathrm{gap,l}}$ denotes the position of the left-hand side of the gap:

$$N_i = \max[-k_g(x_i - x_{\mathrm{gap,l}}) - c_g\dot{x}_i, 0],$$
$$f_i = -\mu N_i \tanh(c\dot{z}_i),$$
$$\mathbf{F}_{\mathrm{reaction},i} = (N_i, f_i)^{\mathrm{T}}.$$

On the other hand, when $x_i > x_{\mathrm{gap,r}}$, $z_i < -x_i + x_{\mathrm{gap,r}}$, and $z_i < 0$ where $x_{\mathrm{gap,r}}$ denotes the position of the right-hand side of the gap:

$$N_i = \max[k_g(x_i - x_{\mathrm{gap,r}}) + c_g\dot{x}_i, 0],$$
$$f_i = -\mu N_i \tanh(c\dot{z}_i),$$
$$\mathbf{F}_{\mathrm{reaction},i} = (-N_i, f_i)^{\mathrm{T}}.$$

We assumed that the horizontal component of the ground reaction force F_i is given by the x component of $\mathbf{F}_{\mathrm{reaction},i}$ considering that each leg is roughly aligned along the z axis.

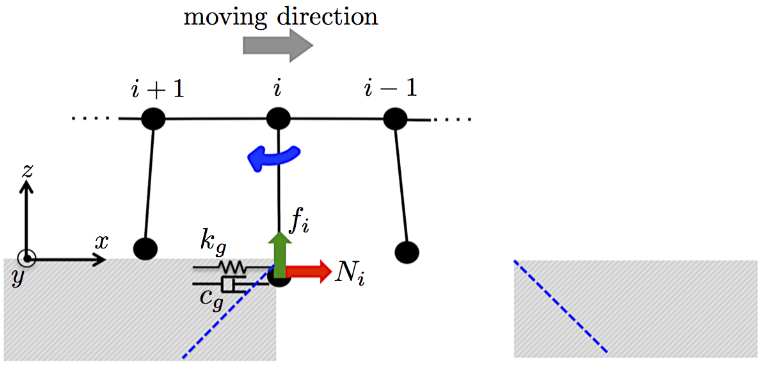

Figure 2. Schematic of the interaction between the legs and side walls.
